# Healthy working in inclusive companies – a study protocol of the GAIN project

**DOI:** 10.1186/s12995-023-00399-x

**Published:** 2023-12-15

**Authors:** Fabian Holzgreve, Bettina Bredereck, Christopher Heim, Britta Weber, Rolf Ellegast, David A. Groneberg, Christian Gaum, Daniela Ohlendorf

**Affiliations:** 1 Institute of Occupational Medicine, Social Medicine and Environmental Medicine, Goethe-University Frankfurt, Theodor-Stern-Kai 7, Building 9a, 60596 Frankfurt am Main, Germany; 2 Department of Psychology and Sports Sciences, Institute of Sport Sciences, Goethe University Frankfurt, Frankfurt am Main, Germany; 3 Institute for Occupational Safety and Health of the German Social Accident Insurance (IFA), Sankt Augustin, Germany; 4 Faculty of Sports Sciences, Ruhr-University Bochum, Bochum, Germany

**Keywords:** People with disabilities, Social firms, Ergonomic risk assessment, Workloads, Workplace design, Workplace health promotion, Inertial motion capture, Inertial motion units

## Abstract

**Background:**

The research project GAIN (working healthy in inclusion companies) deals with the topics of health and work in inclusive companies. Due to a great need for research on (occupational) health (e.g. physical and mental health status) and workplace design in companies employing people with disabilities, this project pursues the primary goal of generating information for the development and implementation of health-preserving measures within the framework of occupational health and safety, and risk assessment, for employees with and without impairments in inclusive companies.

**Methods:**

Within the framework of the project, the employees of three inclusive companies will be examined with the help of an interdisciplinary and triangulative approach. Using quantitative and qualitative methods, specific physical workloads and hazards will be investigated by means of baseline screening methods and measurement techniques, specifically among employees with physical disabilities and impairments. In the statistical analysis, descriptive methods will be used to record the current state, while inferential statistical methods will be used to evaluate health maintenance measures. Inferential statistics for continuous data with confidence intervals based on the statistical parametric mapping (SPM) method will also be performed. The significance level will be set at 5%. Qualitative methods will be used to analyse structures and working conditions within the companies, with particular attention to the specific construction of the relationship between work, health and disability.

**Conclusions:**

The structures in inclusion companies must be specifically designed to support and promote the understanding of work and health in relation to the idea of one’s own body, its positioning in space and its performance. These characteristics are to be identified in the course of the project and bundled into best-practice recommendations. Furthermore, it is the aim of the research project to derive recommendations for action at its conclusion and to present further advice for the promotion of health in inclusive companies.

## Introduction

The claim of an inclusive job market has recently been reaffirmed via the German federal government’s legislative decree [1]. This proves to be congruent with the far-reaching requirements placed on the job market by the UN Convention on the Rights of Persons with Disabilities that officially came into force in Germany in 2009: people with disabilities or health impairments (in the context of this project, this refers to severely disabled people in accordance with §§ 215 et seq. of the German Social Code IX) must be offered the opportunity to take on a job that allows them to earn an adequate living. In order to pursue this objective, numerous support programmes and initiatives have been implemented by politicians in recent years in addition to the adoption of corresponding legal requirements (companies with < 20 employees are obliged to fill 5% of their jobs with people having disabilities). These programmes aim to improve training and employment opportunities for people with disabilities or health impairments, and to strengthen inclusion competencies and inclusion structures among key players in the labour market by raising awareness, providing information and advice [2]. These measures have included the introduction of inclusion companies or integration companies that are legally and economically independent, autonomous companies in which at least 30% severely disabled people, as defined in Sect. 215 of the Ninth Book of the Social Code (SGB IX), are employed.

In 2020, 945 inclusive businesses were recorded in Germany [3] whose focus concerned job security for people with impairments on the one hand and (school) training and subsequent integration into the labour market on the other [3]. The objective of these enterprises is to reduce or eliminate existing “special worlds” at various levels of human interaction. Therefore, in inclusion companies, as in regular companies, the premise of occupational health and safety is to guarantee the health and well-being of all employees through “the conscious control and integration of all operational processes” [4]. This is inextricably linked to the “improvement of work organization and [the] working conditions, as well as the promotion of active employee participation” [5]. In contrast to other companies, inclusion companies have been obliged since 01.01.2018 under §§ 215–218 SGB IX to offer workplace health promotion measures that go beyond occupational health and safety [6].

In addition, severely disabled employees have guaranteed rights that can help to maintain and promote their health, including time off from overtime, additional leave and reduced working hours. In practice, measures are taken for behavioural and situational prevention. Relational prevention measures include the ergonomic design of the workplace which takes into account environmental variables (climate, colour, noise, vibrations, anthropometry) at the workplace. Such hazards due to workloads influence the prevalence of illnesses related thereto. Whether it is possible to apply conclusions drawn from employees without disabilities or health impairments in regular workplaces to employees with disabilities or health impairments in inclusive workplaces remains unclear, (based on the current data situation) since the workplace analyses available to date refer almost exclusively to regular workplaces. Therefore, to summarise, there is a lack of information on the occupational health of employees in inclusive companies.

## Status of the research

Prior to the implementation of the project, an intensive literature search was conducted in both the German and English languages in relevant occupational medicine, psychological and sports science databases (e.g. Pubmed, LIVIVO, bisp-surf) and specialised journals (e.g. Journal of Applied Research in Intellectual Disabilities; Teilhabe Fachzeitschrift der Lebenshilfe) on the topics of workplace ergonomics, health maintenance of employees and barrier-free workplace design in inclusive workplaces and in sheltered workshops. Inclusion criteria were studies that included people with disabilities and adult participants. The search was also limited to projects in Germany. The reason for this was that inclusive companies are defined very differently internationally and the aim of this project was to create a guideline for inclusive companies in Germany. Further search items used were “workplace health promotion” or “WHP” or “health promotion” or “workplace sport” or “health measure” or “health sport” and (“inclusive companies” or “people with disabilities” or “people with impairments” or “workshop” or “Sheltered workshop” or “inclusion”). In English, the search was narrowed to “Workplace Health Promotion” or “Health Promotion” or “Health” and “Social Firms” or “Disability” or “Disabilities” in the advanced “PubMed Search Builder”. The present literature search confirms that there are only a few studies dealing with the implementation and evaluation of health promotion measures in inclusive companies and sheltered workshops. This finding is also shared by Deforche et al. (2018) [7] and Lengen et al. (2021) [6]. However, the studies found by the search differed greatly in their methodologies and objectives.

The project “Fit for inclusion at work” [8] evaluated a health sports programme for mentally disabled people in sheltered workshops. The intervention lasted 18 months and aimed to reduce health risk factors and increase the performance of the participants. The study showed positive effects on cardiometabolic risk, muscle strength and endurance. Different training groups were formed to include a strength training group, a cardiovascular group and a control group. Acceptance of the programmes was high and participation led to a reduction in sick leave days. In developing a guide for low-threshold exercise- and sport-related activation of people with disabilities, Meseck et al. [9] took a comparable approach. Their activation can be conducted without specific instruction or performance measurement and lasts 15 to 20 min. The focus is on short-term activation that can positively influence workplace behaviour. Implementation improves physical, psychological and social skills, as well as the participant’s overall well-being. The guide includes practical examples and recommendations for various games and exercises that differ in their requirement profiles and are suitable for all, e.g. the “dice game”, the “ball caterpillar” and the “pass ball” game. The goal of the short-term activities is a systematic sequence consisting of low intensity activities at the.

beginning that increase during the main game and are then followed by a relaxation exercise afterwards.

In contrast, the “GESUND!” project of the Lichtenberg Workshops in Berlin [10] focussed on health education by raising awareness of health promotion among people with learning difficulties and facilitating joint learning. The project was carried out by a research team from the Catholic University of Applied Social Sciences in Berlin and was funded by the “Prevention Research” programme of the Federal Ministry of Education and Research. The course (health researcher in sheltered workshops) was developed and aimed at disabled employees of the sheltered workshops with an interest in health topics. The curriculum was based on a four-field matrix and allowed for a flexible schedule that was adapted to the needs and interests of the participants. The “rainbow model” of factors influencing health was used to categorise the content developed during the project. The project showed positive results in terms of the participants’ inquisitiveness, the effectiveness of small groups, and the importance of repetition and practise for sustainable learning. The project “GESUND!” also offers an online seminar to teach the topics of heart health, healthy eating and drinking, and relaxation to employees in disability care.

In addition, an analysis of previously funded research projects of recent years between scientific institutes/institutions and companies with inclusive employees shows few results. In the field of logistics, for example, the project “InkluServ” of the Frauenhofer Institute and the WEK Werkstätten Esslingen should be mentioned whose goal is the technological support of electric box bicycles for the transport of goods for an inclusive food market [11].

The Institute of Transmission Technology, Machine Dynamics and Robotics at RWTH Aachen University is working on the design of inclusive human-robot workplaces together with Caritas Wertarbeit (Cologne) and the Fachhochschule des Mittelstands GmbH in one company (12 participants) [12]. In 2017, the Institute for Occupational Medicine, Safety Technology and Ergonomics e.V. (Wuppertal) conducted a research project on an inclusive risk assessment for hearing impaired people in which they developed an inclusive risk assessment [13].

A target-oriented added value for the preservation of employability can be expected in the context of a practice-oriented and occupational safety research, such as best-practice examples of the inclusion award of the German economy in recent years [14]. In addition to national best-practice examples, European or international implementations should also be researched here as these have recently been successfully carried out in an international study by the Federal Ministry of Labour and Social Affairs on accessibility in companies in the private sector [15]. Here, the German Social Accident Insurance (German abbreviation: DGUV) was able to identify successful examples of how access and working conditions for people with disabilities are regulated and improved in the private sector. In addition, the existing offerings of the German Social Accident Insurance Institution for Welfare and Care are to be used as a starting point, and from this extensions and modifications can be made.

These are instruction materials for specific aspects of occupational safety and health (fire protection, tools, internal transport) in an inclusive handout [16]. In this context, a mention should also be made of the guidance on barrier-free work design provided by the German Social Accident Insurance and the technical rules for workplaces issued by the Federal Institute for Occupational Safety and Health (German abbreviation: BAuA), which can be understood as a prerequisite for an ideal inclusive workplace design [17]. Although the projects mentioned show successfully implemented examples from the practice, they do not primarily include the aspect of health maintenance or promotion.

### Aims

The main reason for this lack of research in occupational medicine is probably the relatively small number of inclusive companies in Germany.

In summary, the results of the literature research as well as the deficits in company practice expressed by the executives during the inspections show a great need for research with regard to the recording of the physical and mental state of health, the workplace design and the risk analysis of workloads as well as the design of preventive measures in terms of occupational health and safety in inclusive companies. Due to the special clientele in inclusive companies, it cannot be assumed that common methods from non-inclusive companies for analysing the stress and strain situations at the workplace and for recording the effect of preventive measures are directly transferable. This applies in principle to people with disabilities. However, people without disabilities who work in inclusive companies should also be measured using the same methods so that adjusted target figures allow comparisons to be made. In addition, potential comparison groups from non-inclusive companies should be examined using such adapted methods so that comparisons can be made. Furthermore, there are significant divergences between inclusive and mainstream companies in the range of knowledge and skills of the people working there. This is not only the case with regard to their work competencies, but also, and in particular, with regard to their personal competence in identifying individual stress and strain factors and the ability to describe and articulate them. In order to survey and analyse physical and psychological working conditions, the corresponding risk factors at the workplace [18] and to record the effects of possible measures, it is, therefore, necessary to develop new types of instruments for use in inclusive companies or to modify the existing instruments.

The overall objective of the project is to generate information for the development and implementation of health-preserving measures within the framework of occupational health and safety and risk assessment for employees with and without impairments in inclusive companies. In accordance with the principles of the UN Convention on the Rights of Persons with Disabilities from 2006 (UN-CRPD), people with disabilities, such as managers with disabilities from the participating inclusive companies, should also be involved in the development of interventions and preventive measures. This is intended to avoid an analysis from the perspective of people with disabilities through the eyes of people without disabilities [19]. From this, concrete advice on work design is to be derived for inclusive companies as well as for regular companies that wish to increase their share of employees with disabilities or health impairments. On the one hand, the relevance of the topic is to be sensibilised and, on the other hand, regular companies can be encouraged to employ people with disabilities or health impairments. These objectives are to be achieved in several research steps with superordinate questions, setting the following focal points:

1. Survey of the current state:

- What risks to the physical and mental health of employees with and without disabilities or health impairments occur at workplaces in inclusive companies?
- Is it possible to differentiate between stress resulting from incorrect work-related adaptation and stress resulting from the employee’s own disability?

To answer these questions, conspicuous types of physical stress should first be identified using the DGUV checklist (orienting risk assessment for stresses on the musculoskeletal system). If conspicuous physical stresses are present, a differentiated ergonomic risk assessment of selected key stress points is to be carried out using measurement techniques based on inertial sensors (Xsens Motion Capture, MVN Awinda) in combination with other sensors (e.g. heart rate measurement). In line with the mixed-methods design, exploratory observational studies, guided interviews, and questionnaire surveys will also be conducted.

Ethnographic observations: the procedures and requirements of the work process; structures, communication processes and management style of inclusive companies.

Interviews will ask about experiences and impressions of work activity (e.g., stresses, strains, routines, organization, social hierarchies) and health (e.g., well-being, pain).

2. Development of measures:

- To what extent are the existing questionnaires for recording complaints in the musculoskeletal system, interview and observation techniques, and measurement techniques suitable for implementation in the selected inclusive pilot companies, or what adaptations may be necessary for implementation in inclusive companies?
- To what extent are selected prevention measures and concepts of non-inclusive workplace design suitable for implementation in the selected inclusive pilot companies, or what adaptations may be necessary for implementation in inclusive companies?
- What effects can be achieved by the application of appropriately adapted measures in inclusive companies on the health maintenance of the employees?

To answer these questions, the effectiveness of ergonomic interventions in the respective work processes will be examined with the aid of a differentiated ergonomic risk assessment. These measurement methods will be complemented by qualitative interviews. In principle, it must be taken into account that due to the special nature of the field, a corresponding methodological adaptation will be necessary.

3. Implementation of the measures and knowledge transfer:

- How can the modified or newly developed methods be transferred to the assessment of the health and workload of people with disabilities or health impairments?
- How can the knowledge gained be translated into long-term concepts of inclusive workplace health maintenance?

The aim of this project, therefore is, to ensure that the results are linked to practice by anchoring them in concepts for health maintenance and promotion. For this purpose, handouts for inclusive companies and, if necessary, also regular companies will be created. This knowledge transfer not only refers to the findings on the requirements and starting points for inclusive workplace health promotion, but also to the modified and newly developed methodological approaches and adapted research instruments (e.g. the availability of the Nordic Questionnaire in easily accessible language).

## Materials and methods

### Subjects

Within the scope of this project, employees of various inclusive businesses will be examined. These businesses include a laundry service with 122 employees, a company in the field of office services and document management with 64 employees and a fitness and health centre with about 15 employees. The proportion of people with disabilities in the inclusive companies is between 43% and 48%. Furthermore, employees of a sheltered workshop with 337 employees (approximately 91% with disabilities or health impairments) will be analysed.

In the laundry service and fitness and health centres, the employees mainly work in a standing position, e.g. on an assembly line, while those in the office services and document management, as well as in the sheltered workshops, mainly work in a sitting position.

The following inclusion and exclusion criteria apply:

Inclusion criteria:

- Employees of the companies mentioned.
- People with and without disabilities (physical and mental disabilities, i.e. deafness, learning disorder or employees returning to work after serious illness or injury).
- Voluntary participation, declaration of consent.
- Participants have full legal capacity and understand the nature, scope, significance and consequences of the examination.
- No current injuries of the musculoskeletal system.

Exclusion criteria:

- Intake of perception-altering substances.
- Acute injuries.

A signed consent form will be obtained from all participants. This form includes comprehensive information about the objectives and the course of the study as well as personal rights and data protection. The above-mentioned aspects will be discussed together in a personal interview and on the informed consent form. Due to the heterogeneity of the participants, the clarification will be adapted to the individual challenges (comprehension problems e.g. due to cognitive, intercultural characteristics) (e.g. easy language).

An approved ethics application from the Department 05 of the Goethe University Frankfurt (2022-57) is available for the implementation of the studies.

### Risk analysis

#### DGUV checklist

The risk analysis should be carried out using the “Checklist for orienting risk assessment for musculoskeletal system loads” from DGUV Information 208 − 033 [20]. The DGUV checklist enables a quick and simple assessment of the physical types of stress that can be used to identify focal points of stress. It also provides an orienting clarification of the question of whether there is a need for action, e.g. an in-depth analysis or measures to be taken to reduce the load.

### MVN Awinda

The MVN Awinda motion capture system is a person-based system that uses 17 inertial motion sensors, each consisting of accelerometers, gyroscopes and magnetic field sensors, to provide positional or angular information of the entire human body. The system’s sampling rate is 60 Hz and the measurement error is specified by the manufacturer as ± 1%. The measurement system works wirelessly and the data is transmitted by radio. The sensors are attached to the body parts with the help of adhesive tape. In contrast to the MVN Link system in which wired sensors are attached to a full-body suit, this method offers a large degree of flexibility in handling so that individual and anthropometric differences can be optimally taken into account, especially before working with people with disabilities. In contrast to the gold standard optical motion tracking, this inertial motion capture system provides reliable data, ranging from good to excellent, in line with concurrent research, particularly in the frontal and sagittal planes [21, 22].

### Ethnographic observations

Observational studies will be carried out in the selected companies in order to elicit the procedures and requirements of the work processes using a descriptive approach. All persons present in the company will be informed about the aims and the course of the ethnographically designed observations. The observations do not collect personal data but refer to the work processes themselves. They are divided into two parts. The first one contains observations with immediate field notes, that are added by memos afterwards. During the second part the observations are made by accompanying one employee for a few hours. This employee is questioned about the work he or she is doing. Field notes and memos are prepared afterwards.

### Guideline-based interviews

Guided interviews will be conducted with the management of the participating companies. These interviews are oriented along three thematic blocks relating to basic structures, inclusive companies and management style. The aim is to obtain an initial overview of the company and management structures and to discover which company health measures are or were a regular part of everyday working life. Following the observations, guided problem-centred interviews will be conducted with selected employees of the companies. In this way, it is possible to learn more about the subjective views of the employees.

Especially in interviewing mentally disabled people, the use of standardised methods validated via tests with non-disabled people is often limited because the possibilities to flexibly respond to the individual relevance criteria of the respondents are reduced with the increasing standardisation of the survey [23]. In general, follow-up questions and interpretation are necessary and misunderstandings have to be excluded. The basic prerequisite for surveys with mentally handicapped people is, therefore, a precise knowledge of the life situation of the interlocutor and the illustration of what is meant in a form that the interlocutor can understand [24]. This results in the focus of a subject-oriented approach in combination with an ethnographic approach. However, this makes it absolutely necessary to first get to know the persons to be interviewed personally in order to establish a basis of trust and to clarify the meaning and purpose of the research [25]. The selection is based on the specifics of the activity, personal data (type of impairment) and the willingness to participate. Interviews will ask about experiences and impressions of work activity (e.g. stresses, strains, routines, organisation, social hierarchies) and health (e.g. well-being, pain). In the interviews, personal (e.g. motivation, wishes), structural (e.g. tasks, scope, work equipment) as well as social content areas (e.g. interaction with colleagues) are to be made the subject of discussion. The interview data is transcribed by a service provider. The evaluation will be based on the Grounded Theory [26, 27].

### Questionnaire of musculoskeletal complaints

In order to be able to analyse the health of the employees more comprehensively for the recording of potential correlations between surveyed work-specific physical stresses and physical complaints or pain of the employees, a quantitative survey of physical complaints or pain is to be carried out by means of a questionnaire in addition to the qualitative survey of health. Furthermore, personal data such as height, weight, age, gender and the types of any limitations will be collected via the questionnaire and subsequently pseudonymised.

Since it is not possible to refer to questionnaires validated “in the inclusion setting”, the aim is to develop and validate a new questionnaire. In the development of the questionnaire, the focus was mainly on elements from the Nordic Questionnaire [28]; this questionnaire has been an internationally recognised and widely used questionnaire for the assessment of musculoskeletal complaints for more than 30 years. It has been validated several times [17, 29, 30] and there are comparative data available from administrative occupations [31–34], factory workers [35–38] or employees in health care occupations [39–42]. In this project, the revised German version of the Nordic questionnaire on musculoskeletal complaints [43] from the Federal Institute for Occupational Safety and Health serves as a template. Furthermore, in addition to elements of the Nordic Questionnaire, elements from the German Pain Questionnaire are also to be used which appear to be simpler and easier to understand for implementation by people with disabilities.

Besides the translation into easy language, the adaptation to the target group’s understanding of the text (especially regarding the meaning of pain) is mandatory. For this purpose, participatory interviews on the questions of the questionnaire are planned. Validation will be carried out via piloting with selected people with disabilities.

### Study procedure

The plan is to conduct an exploratory baseline study in the companies listed above, focussing on their respective professional activities. Job requirements include sedentary desk work in an office or gym, fine motor work in a sitting or standing position in a sheltered workshop, repetitive tasks performed in a standing position in assembly work, or physically intensive work in physical therapy. A triangulated combination of qualitative and quantitative methods (mixed-methods design) will be used to record the physical and mental health burden. From the outset, a participatory approach will be pursued in which the research will not be conducted about, but rather with, those affected, in close accordance with DGUV Information 215 − 112 [17].

First of all, the literature research is to be extended since the previous literature research did not reveal any or very few relevant studies on health measures in inclusion companies and workshops for people with disabilities. In this context, already successfully implemented improvements of workplace situations (in the form of best-practice examples) are to be identified.

Initial workplace inspections and surveys of the occupational safety and health stakeholders are planned on site in the inclusive companies in order to obtain initial findings with regard to physical and mental stressors, other risk factors and resistance resources in the inclusive context that achieve productive and preventive effects.

The identification of increased physical stresses for the musculoskeletal system is to be carried out using coarse screening (DGUV checklist [20]). The DGUV checklist provides an orientational risk assessment in the six physical load types of lifting, holding and carrying loads, pulling and pushing loads, manual work processes, exertion of whole-body forces, forced body postures and body continuation. In addition, hand-arm and whole-body vibration will be examined. In order for the coarse screening to be applied in a meaningful way, it is first necessary to learn about and understand the overall work processes in the participating companies. In this way, the manual activities of the respective workstations are to be objectified and potential risk factors identified within the framework of job observations and surveys of potential ergonomic risks.

The DGUV checklist is then to be applied in selected work steps to identify the types of exposure and rough exposure levels. For this purpose, the investigators will take on an observing role and carry out the analysis by observing the activities while the study participants are carrying them out undisturbed.

If increased loads and, thus, potential hazards are identified, then this will be followed by a differentiated ergonomic risk assessment of selected load focal points of the measurement data collected by means of motion capture procedures based on inertial sensors (MVN Awinda). The exposure assessment will be carried out using established occupational science and biomechanical procedures, both from the category of screening procedures, in which a rough assessment of types of stress is carried out (e.g. rapid upper limb assessment (RULA) [44], key indicator methods and the DGUV checklist from the MEGAPHYS project [45]), and from the category of measurement procedures which allows a localisation-specific risk assessment of the continuously collected measurement data (CUELA assessment procedure, MEGAPHYS [45, 46]). To record possible subjective complaints in the musculoskeletal system, a modified questionnaire (variations of the Nordic Questionnaire) was designed and evaluated specifically for the needs of the employees (easy language, supportive communication). This questionnaire will make it possible to identify correlations between subjectively perceived complaints and objectively measured stresses in specific body regions. Following validation, the questionnaire will then be used for surveying the employees of the cooperating companies. Translators will accompany the practical implementation if, for example, non-native speakers are interviewed or sign language is required.

In addition, participatory observations lasting several weeks are to be carried out in order to gain in-depth insights into everyday work processes and to establish personal ties with the employees as a prerequisite for a subsequent survey. Based on DGUV Information 206 − 026 on mental stress [47], this will be implemented via problem-centred interviews and also within the framework of participant observations (interview notes, memos, etc.). The survey is intended to record the subjective well-being at the workplace. For this purpose, aligned guideline-based interviews are planned which are to be developed and used in this phase. The planning and implementation of the analyses is to be carried out in close coordination with the occupational health and safety actors.

Based on the results of the workplace analysis and the survey of the physical and mental state of health, conclusions are to be drawn about potential health hazards for the employees. In addition, specific workplaces are to be identified in which exemplary measures can be implemented. These measures will then be implemented in consultation with the respective plant management and with the involvement of the employees. Possible starting points here could be the ergonomic redesign of workplaces (work equipment, work furniture, room concept, noise and lighting conditions), target group-appropriate instructions for healthy behaviour (e.g. video clips on chair adjustment) or the visualisation of body movements and postures (e.g. via Xsens or CUELA). The objective here is to examine whether and to what extent proven concepts from non-inclusive workplaces can be transferred to inclusive workplaces or which adaptations of these concepts are necessary for use in inclusive workplaces. The implementation and evaluation of the implemented measures will be surveyed in the respective inclusion companies over a period of approximately 6 months (pre-post control group design). The results of the evaluation will be used to draw conclusions about the applicability of the modified analysis methods and the transferability of the implemented intervention measures (transfer of best-practice examples). Recommendations for action are to be derived from the findings with the aim of achieving a high degree of transferability and easy implementation in operational practice. Since this project focusses on the analysis of “classic” occupational activities (office work/assembly work/stand-up work), which often correspond to the work requirements in inclusive companies a high degree of transferability to other companies can be assumed.

### Evaluation parameters

#### DGUV checklist

The presence of an increased load in one or more of the examined load types.

### Measuring analysis

The motion data acquired with MVN Awinda will be used to analyse the load for the body regions neck/cervical spine, shoulders/upper arms, elbows/lower arms, wrists/hands, lower back/lower spine, hips and knees, as well as to analyse the cardiovascular system and energy metabolism, and to evaluate all these with a view to possible health hazards. For this purpose, the movement data will be further processed with proprietary software [45, 47] to determine the relevant biomechanical and physiological load parameters over time:

- Body angles and angular velocities (e.g. tilt/lateral tilt/torsion of the head and trunk, flexion in the knee, hip, shoulder, elbow and wrist joints).
- Estimation of joint moments (e.g. shoulder joint or lumbar spine at L5/S1).
- Estimation of intervertebral disc compression forces in the lumbar spine in the L5/S1 region.
- Working heart rate.
- Energy metabolism.

The assessment of the exposure data will be based on:

- ISO standards.
- RULA score [45].
- Body region-related CUELA assessment procedures [47].

*Questionnaire*.

− 12 months’ prevalence and frequency per body region.

- Duration of complaints.
- Influence of profession.
- Cause of complaint.

### Interviews

The survey method of guided interviews will be used to collect data. The subsequent evaluation will be carried out with the help of Grounded Theory.

The interviews with the companies’ managements will be collected with the help of the procedure of problem-centred interviews and the Grounded Theory, and will be evaluated via the categories of basic structures, inclusion companies (tasks and duties regarding WHP) and management style. The guideline for the interviews with the employees of the companies follows the deductive structuring into work processes, experiences and specific conflicts and adaptations to the work requirements and the employees’ own handling of health or physical complaints. In addition, the self-image of inclusive companies and the perception of hierarchical structures are addressed in accordance with the survey of the management level.

Additionally, the interviews also serve to involve people with disabilities in the process. Through the interviews they are able to share their subjective view on (dis)ability, the workplace and health. In this manner every other part of the research process includes these statements in their solution finding process.

### Statistical analysis and sociological evaluation of the interviews and observations

The project intends to do this in accordance with an inclusive understanding, together with the greatest possible openness to the needs of the people working there. For this reason, a precise indication of the sample size is not yet possible, at this point, as participation can only be voluntary. In order to win over as many of the employees as possible for participation, a detailed explanation to the employees will take place, in consultation and cooperation with the BWMK works council, before the data collection begins. The aim (which is, according to the “Behinderten Werk Main-Kinzig” interviewees, a realistic one) is for at least 50% of employees to participate.

### DGUV checklist

This is a dichotomous parameter that is used to assess roughly the physical risk. Only descriptive methods are used for this purpose.

### Measurement analysis

After processing the kinematic data with the Xsens software, the joint angle data are further processed in user-defined files using MATLAB® software vR2020a (The Mathworks Inc., Natick, MA, USA). For ergonomic risk assessment of the recorded kinematic data, the RULA algorithm was applied using a code developed by Maurer-Grubinger et al. [44]. Further details on this script can be found in Maurer-Grubinger et al. [44].

In order to compare the joint positions or joint angles during the examination period, an inferential statistic for continuous data will be used. This statistic is based on the application of confidence intervals and uses the statistical parametric mapping (SPM) procedure.

All test procedures to be used are two-sided and are subject to a 5% significance level.

### Questionnaire

The survey questions consist of nominal and ordinal scales. Descriptive and inferential statistical methods will be applied for the statistical analysis. The Chi-square test will be used for testing differences, with a significance level of 5%. Since both individuals with and without disabilities perform the same occupational activities in the participating companies, a direct workplace comparison is possible using all the survey methods employed.

### Interviews and observations

The qualitative data from the interviews and observations will be analysed based on the Grounded Theory approach using a multi-stage coding process. Due to the integration of qualitative and quantitative methods, the theory formation is not entirely inductive and theory-free. However, given the exploratory nature of our study design, this approach is considered appropriate. The observations, development of guidelines, conduct of the interviews and analyses will be carried out in a circular and non-linear manner, as is common in qualitative research.

The basic step is initially the Open Coding which involves an initial, unbiased review. Categories resulting from this step are analysed in the second step, Axial Coding, for their interconnected meaning. In the third step, which involves further abstraction, initial hypotheses can be formed, thus leading to broader conclusions. Furthermore, the categories will be continually summarised throughout the process, ultimately crystallising into key categories. The inherent intention of identifying a core category in the theory will not be pursued due to the diverse focal points of the research questions.

## Discussion

In contrast to regular businesses, inclusion companies are obligated, according to §§ 215–218 SGB IX since 1 January 2018, to offer occupational health promotion measures beyond occupational safety and health protection [6]. However, existing workplace analyses mostly focus on regular businesses, while detailed analyses of the prevalence of physical and mental hazards among employees with disabilities or health impairments are lacking. This also applies to systematic insights into the structural conditions or health preservation measures in inclusion companies [2]. This deficiency begins with the collection of commonly used metrics in regular businesses. For example, information about the number of disability-related sick days due to physical and mental complaints is not transparent, making it unknown whether these differ from the general population [48]. However, some findings do exist in general medical research; for instance, people with disabilities or health impairments show a higher prevalence of musculoskeletal disorders (MSD) compared to those without [49, 50]. In addition, risk factors for metabolic syndromes and physical inactivity are more pronounced in individuals with intellectual disabilities [51]. As a result, health preservation measures for individuals with disabilities or health impairments generally focus on behavioural prevention, concentrating on physical activity programmes [52] and the promotion of sports activities [53]. Studies that analyse the potential impact of population-level prevention measures for people with disabilities or health impairments are not known, neither nationally nor internationally.

The current project’s approach follows an interdisciplinary and triangulated approach. From an occupational health perspective, specific workloads and hazards will be investigated using quantitative methods, especially for individuals with physical disabilities and impairments. From a social science perspective, the qualitative methods will focus on the structures and working conditions within the companies, examining the specific interplay between work, health and disability. Additionally, qualitative methods permit, due to their methodological approach, the presentation of subjective views and life experiences of people with disabilities. Therefore, it is possible to include these subjective perspectives and individual strategies in the solution finding process of the entire research project. It is important to note that the planned analyses will always prioritise the specific occupational activity (e.g. sedentary desk work, fine motor work sitting or standing in a workshop, standing while carrying out repetitive tasks in assembly work). These three types of activities are generalisable and potentially applicable to various companies.

With the help of the findings from the planned study, solutions can be developed for the first time in the form of health-preserving workplace design options for employees with disabilities or health impairments in inclusive companies. It is worth highlighting the expected dual transferability of the analysis methods and adapted health preservation interventions from the non-inclusive sector to the inclusive. Furthermore, the project aims to present health promotion offerings to inclusion companies. Concrete measures of behavioural prevention could include diverse sports and health programmes as well as extensive training opportunities (e.g. exercise, relaxation and informational offerings), however, these activities are mostly situated outside or complementary to work activities. Individual population-level preventive measures should also be considered, such as an ergonomic workplace assessment (increasing accessibility and, consequently, improving the work situation, which could potentially lead to a reduction in MSD). Notably, practical implementation of health-preserving measures is particularly anticipated from employees with disabilities or health impairments and can directly influence the operational practices of inclusion companies. Pursuant to DGUV Information 215 − 112 [17], achieving maximum accessibility is the primary goal. Based on this information, the intention is to derive future design possibilities for the work environment and workplaces from the specific needs of individuals with disabilities or health impairments and the local conditions.

From a psychological perspective, this could lead to increased work motivation and positive effects on the personal life situation of employees which, in turn, can positively impact social environmental contexts (social dimensions) as well as internal operational processes (economic dimensions). The long-term effects of improved workplace design are expected to positively influence both the maintaining of employability until retirement and the development of the work atmosphere. There is a high probability that these aspects of health preservation will apply to both groups of employees (those with and without disabilities or health impairments). Through healthy employees, increased workplace safety and a low number of work absences, the company also gains to benefit (e.g. increased productivity).

Furthermore, the project aims to consolidate best-practice recommendations and derive action recommendations from the findings. This can contribute to “positive examples” to improve workplace safety and overall work and employability, especially for people with disabilities, which could, potentially, lead to cost reductions for the German pension and health insurance systems. Conversely, as systematic insights into structural conditions or health preservation measures in inclusion companies are lacking, and workplace analyses and data on the prevalence of physical and mental hazards among employees mostly focus on regular businesses, some limitations need to be considered in the implementation of these recommendations. In particular, data collection for individuals with disabilities or health impairments poses extremely high demands on both the applied research methodology and the data-collecting individuals who must possess a high degree of empathy as well as respect, appreciation and authenticity for their subjects. Hypothetically, it must also be considered that after analysing the current state, no intervention options or need for interventions regarding a health-preserving redesign of individual workplaces may be identified. In this case, the project would be paused at this point and further steps would be re-evaluated. Furthermore, a quantitatively designed questionnaire for use with people with disabilities has limited validity for its general application in inclusion companies due to the wide range of disabilities and degrees of disability of the employees. Therefore, the design of the questionnaire for capturing musculoskeletal complaints in this project is tailored to the disability spectrum and degree of disability of the employees in the participating inclusion companies and may not, necessarily, be applicable to all inclusion companies. It is important to note that while the questionnaire is formulated in plain language, not all employees may be able to answer it according to the validation criteria due to the nature and severity of their disability. This is especially important to consider for future applications in sheltered workshops as a higher degree of severe disability typically exists in these establishments.

## Conclusion

The structures in inclusion companies must be specifically designed to support and promote the understanding of work and health in relation to one’s own body, its positioning in space and its performance. This includes creating an inclusive and barrier-free work environment that considers the individual needs and abilities of the employees. Within the framework of the project, these features will be examined over time and consolidated into best-practice recommendations to establish a foundation for designing health-promoting measures in inclusion companies.

Furthermore, the primary goal of the research project is to derive concrete action recommendations. These recommendations are intended to assist inclusion companies in implementing suitable measures to promote the health of their employees. Moreover, the project aims to present further offerings and methods for health promotion to inclusion companies. This could include the development of training programmes for staff, the implementation of ergonomic workplace solutions, the advancement of methods for assessing health and stress, or the integration of health and fitness activities into the work routine. By providing such offerings and methods, the health and well-being of employees in inclusion companies are expected to be improved in the long term.

## Data Availability

Not applicable.

## List of abbreviations

SGB IX: Ninth Book of the German Social Code
GAIN: working healthy in inclusion companies
SPM: statistical parametric mapping
DGUV: German Social Accident Insurance
BAuA: Federal Institute for Occupational Safety and Health
RULA: rapid upper limb assessment
MSD: musculoskeletal disorders
